# Genome-wide assessment of post-transcriptional control in the fly brain

**DOI:** 10.3389/fnmol.2013.00049

**Published:** 2013-12-09

**Authors:** Shaul Mezan, Reut Ashwal-Fluss, Rom Shenhav, Manuel Garber, Sebastian Kadener

**Affiliations:** ^1^Biological Chemistry Department, Silberman Institute of Life Sciences, The Hebrew University of JerusalemJerusalem, Israel; ^2^Program in Bioinformatics and Integrative Biology, University of Massachusetts Medical SchoolWorcester, MA, USA

**Keywords:** post-transcriptional regulation, RNA-sequencing, polyA tail, *Drosophila melanogaster*, brain, miRNA

## Abstract

Post-transcriptional control of gene expression has central importance during development and adulthood and in physiology in general. However, little is known about the extent of post-transcriptional control of gene expression in the brain. Most post-transcriptional regulatory effectors (e.g., miRNAs) destabilize target mRNAs by shortening their polyA tails. Hence, the fraction of a given mRNA that it is fully polyadenylated should correlate with its stability and serves as a good measure of post-transcriptional control. Here, we compared RNA-seq datasets from fly brains that were generated either from total (rRNA-depleted) or polyA-selected RNA. By doing this comparison we were able to compute a coefficient that measures the extent of post-transcriptional control for each brain-expressed mRNA. In agreement with current knowledge, we found that mRNAs encoding ribosomal proteins, metabolic enzymes, and housekeeping genes are among the transcripts with least post-transcriptional control, whereas mRNAs that are known to be highly unstable, like circadian mRNAs and mRNAs expressing synaptic proteins and proteins with neuronal functions, are under strong post-transcriptional control. Surprisingly, the latter group included many specific groups of genes relevant to brain function and behavior. In order to determine the importance of miRNAs in this regulation, we profiled miRNAs from fly brains using oligonucleotide microarrays. Surprisingly, we did not find a strong correlation between the expression levels of miRNAs in the brain and the stability of their target mRNAs; however, genes identified as highly regulated post-transcriptionally were strongly enriched for miRNA targets. This demonstrates a central role of miRNAs for modulating the levels and turnover of brain-specific mRNAs in the fly.

## Introduction

Steady-state levels of mRNAs are a consequence of a balance between transcription and degradation rates. Work done in this area in the last few decades has demonstrated that mRNA molecules are subjected to post-transcriptional regulation of different kinds. These modes of regulation include among others deadenylation, stabilization or degradation by RNA-binding proteins, nonsense-mediated decay reduction (NMD), and miRNA-mediated regulation (Bevilacqua et al., 2003; Alonso, 2005; Halbeisen et al., 2008; Wen and Brogna, 2008; Brogna and Wen, 2009; Meisner and Filipowicz, 2010; Braun et al., 2012). Post-transcriptional regulation usually impacts mRNA stability by influencing or determining the degradation rate. In these cases, cellular control over steady-state levels is achieved mainly by tight post-transcriptional regulation mechanisms rather than by regulating the transcription rate *per se*.

Although several studies have comprehensively assessed post-transcriptional control and RNA turnover rates, these assessments have been restricted either to unicellular organism e.g.,(Andersson et al., 2006; Shock et al., 2007; Miller et al., 2011; Morey and Van Dolah, 2013; Rustad et al., 2013) or cells in culture (Filipowicz et al., 2008; Sharova et al., 2009; Rabani et al., 2011). In other cases, turnover rates have been extrapolated by comparing the levels of nascent and total RNA levels. Although powerful, this type of methodology requires large amount of material and/or laborious procedures (Core et al., 2008; Menet et al., 2012; Rodriguez et al., 2013).

Post-transcriptional regulation of mRNA stability and decay is dictated mainly by trans-acting factors like miRNAs, siRNAs, and RNA binding proteins. These factors act on *cis* elements usually located in the 3′ untranslated region (UTR) of the target mRNA [e.g., AU rich elements, miRNA binding sites (Chen and Shyu, 1994; Kai and Pasquinelli, 2010)]. Their mode of action involves the direct or indirect recruitment of the mRNA degradation machineries like deadenylases, decapping enzymes, and the exosome complex, (for review see Houseley and Tollervey, 2009). A major/convergent point of control on mRNA stability is the length of the polyA tail. Indeed, most pathways that control mRNA turnover affect directly or indirectly the length of the polyA tails (Fabian et al., 2010; Huntzinger and Izaurralde, 2011).

MiRNAs are small (20–23 nucleotide) non-coding RNAs that serve as post-transcriptional regulators of gene expression (Bartel, 2009). MiRNAs are produced in two sequential cleavage steps by the microprocessor complex and the RNAse III enzyme *dicer* (Denli et al., 2004). Their mechanism of action involves the formation of imperfect hybrids with 3′ UTRs of target mRNAs, which results in translational repression, recruitment of the deadenylase GW182, and mRNA degradation (Fabian et al., 2010; Huntzinger and Izaurralde, 2011). miRNAs associate with the target mRNA as part of a large silencing complex called RISC, which in *Drosophila* includes the protein AGO-1 (Bartel, 2009).

Control of mRNA stability has a central importance in the brain: local translational control and mRNA degradation and stabilization in response to changes in neuronal function and activity are critical for proper brain function. Indeed many RNA-regulators (miRNAs and RNA-binding proteins) are important actors in behavioral processes (Kadener et al., 2009; Liu et al., 2012; Luo and Sehgal, 2012; Lim and Allada, 2013; Zhang et al., 2013) and neuronal function in general. Moreover, miss-regulation of RNA stability can lead to neuronal-related pathologies (Aw and Cohen, 2012; Liu et al., 2012). Despite the importance of post-transcriptional control in the brain, no studies to date have globally assessed mRNA stability and the extent of post-transcriptional control in this tissue.

In this study, we performed a genome-wide assessment of post-transcriptional control in the fly brain. We did so by comparing the levels of polyA-selected and rRNA-depleted RNA samples. As rRNA-depleted RNAs include both nascent and unstable RNAs, for a given transcript the relative amounts between the rRNA-depleted and polyA selected samples is a surrogate of the amount of post-transcriptional control and should be inversely related to the stability of this mRNA. We validated our results by showing that, first, housekeeping genes (like those encoding ribosomal proteins and key metabolic enzymes) are the most stable mRNAs identified using our approach and, secondly, that the mRNAs under the control of the circadian clock, and hence expected to have high turnover rates are actually enriched among the less stable transcripts according to our prediction. Interestingly we found that mRNAs ranked as highly stable or unstable are enriched for genes with very specific Gene Ontology (GO) categories. In particular, mRNAs encoding proteins related to neuronal function and physiology are strongly enriched among the less stable mRNAs. Moreover, we found that the mRNAs predicted to be highly regulated post-transcriptionally by our criteria, are highly enriched for miRNA binding sites. In order to determine whether specific miRNAs mediate most of this regulation, we profiled miRNA expression in the *Drosophila* brain using oligonucleotide miRNA microarrays. Surprisingly, we did not find a correlation between the level of expression of miRNAs in the *Drosophila* brain and the extent of post-transcriptional control of the predicted targets. This demonstrates that although miRNAs have a central function in regulating brain mRNAs, the regulation likely involves many layers and complex mechanisms.

## Results

### Use of the polyA plus to total rna ratio to assess global mRNA stability

In a recent study, Hughes et al., generated RNA-seq data from rRNA-depleted RNA (also called total RNA, TR) and polyA+ RNA (PA) isolated from fly brains (Hughes et al., 2012). Contrary to polyA+ RNA, rRNA-depleted RNA includes all forms of RNA, among them nascent RNAs (pre-mRNA) and RNA with short (or no) polyA tails. Hence, transcripts with strong post-transcriptional control would be more enriched in this preparation than in the polyA+ RNA fraction. Therefore, we reasoned that for a given mRNA, the ratio between the abundance in the TR and the PA libraries should be proportional to the amount of post-transcriptional control. A low PA/TR signal indicates strong post-transcriptional control: mRNAs with short polyA tails tend to be found more abundantly in the total RNA fraction, as these transcripts bind weakly to the oligo dT beads used to isolate polyA+ mRNAs (Meijer et al., 2007; Meijer and de Moor, 2011; Kojima et al., 2012).

**Figure 1:**
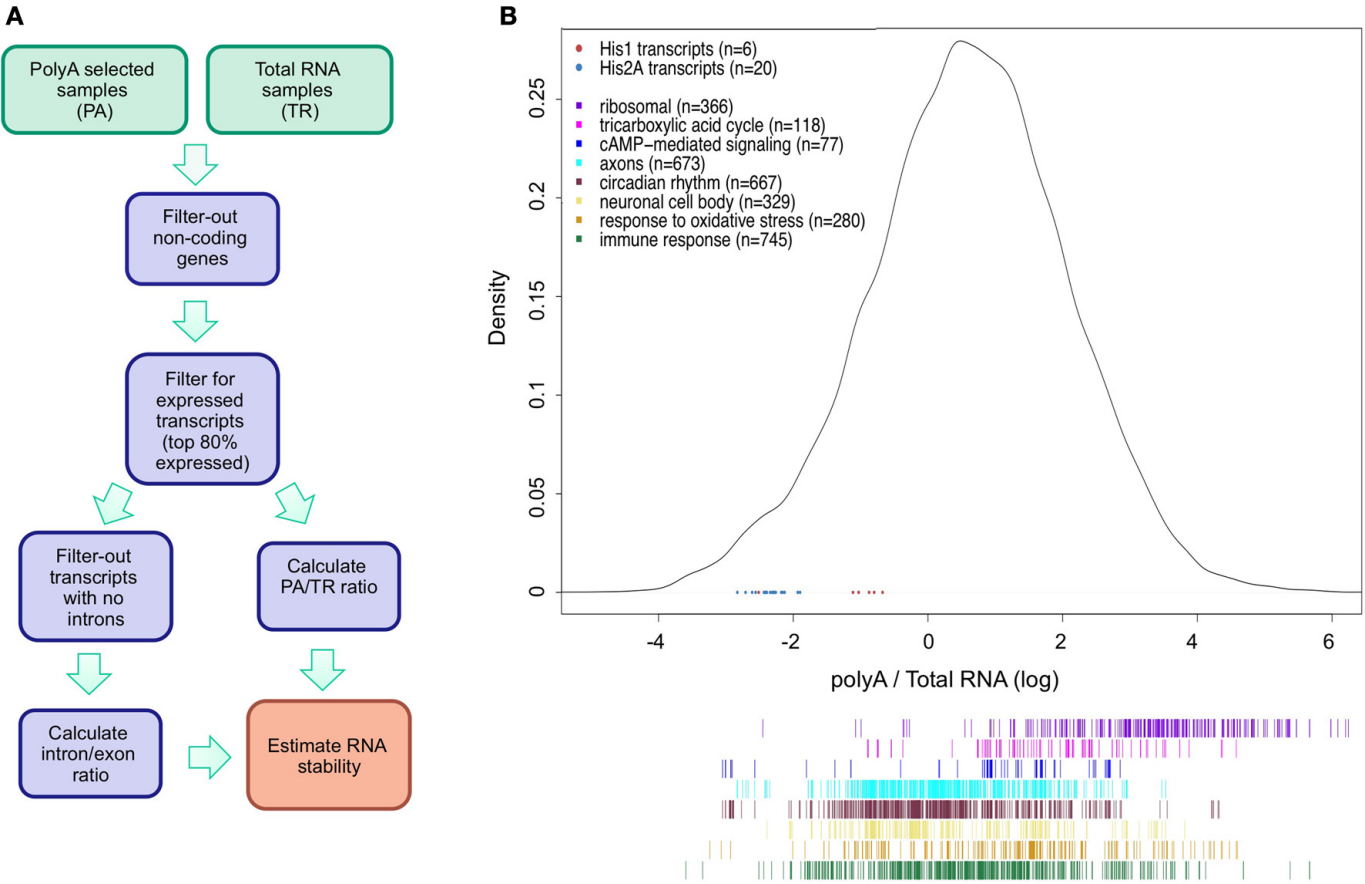
**Analysis of mRNA stability using polyA and total RNA-seq data. (A)** Schematic illustration of the strategy used to estimate RNA stability. **(B)** Density plot of the distribution of polyA/total-RNA (PA/TR) values for all transcripts (*n* = 32898). Data was log-transformed to achieve normal distribution. Blue and red dots at the lower part of the plot represent the PA/TR values of His1 (red) and His2A (blue) transcripts. Colored lines under the density plot represents transcripts associated to different GO terms groups. Color Key for Histone and GO terms transcripts is presented at the top left panel.

We limited our analysis to the transcripts produced by RNA polymerase II as RNAs transcribed by other polymerases lack a polyA tail and hence will only be present in the TR fraction. The data was processed as indicated in Figure 1A. As shown in Figure 1B, the data have a quasi-normal distribution after log transformation (*n* = 32898). As expected, transcripts that are not polyadenylated, such as some of the histones transcripts, are toward the left end of the curve as these have low PA/TR ratios (Figure 1B, red and blue dots).

**Figure 2:**
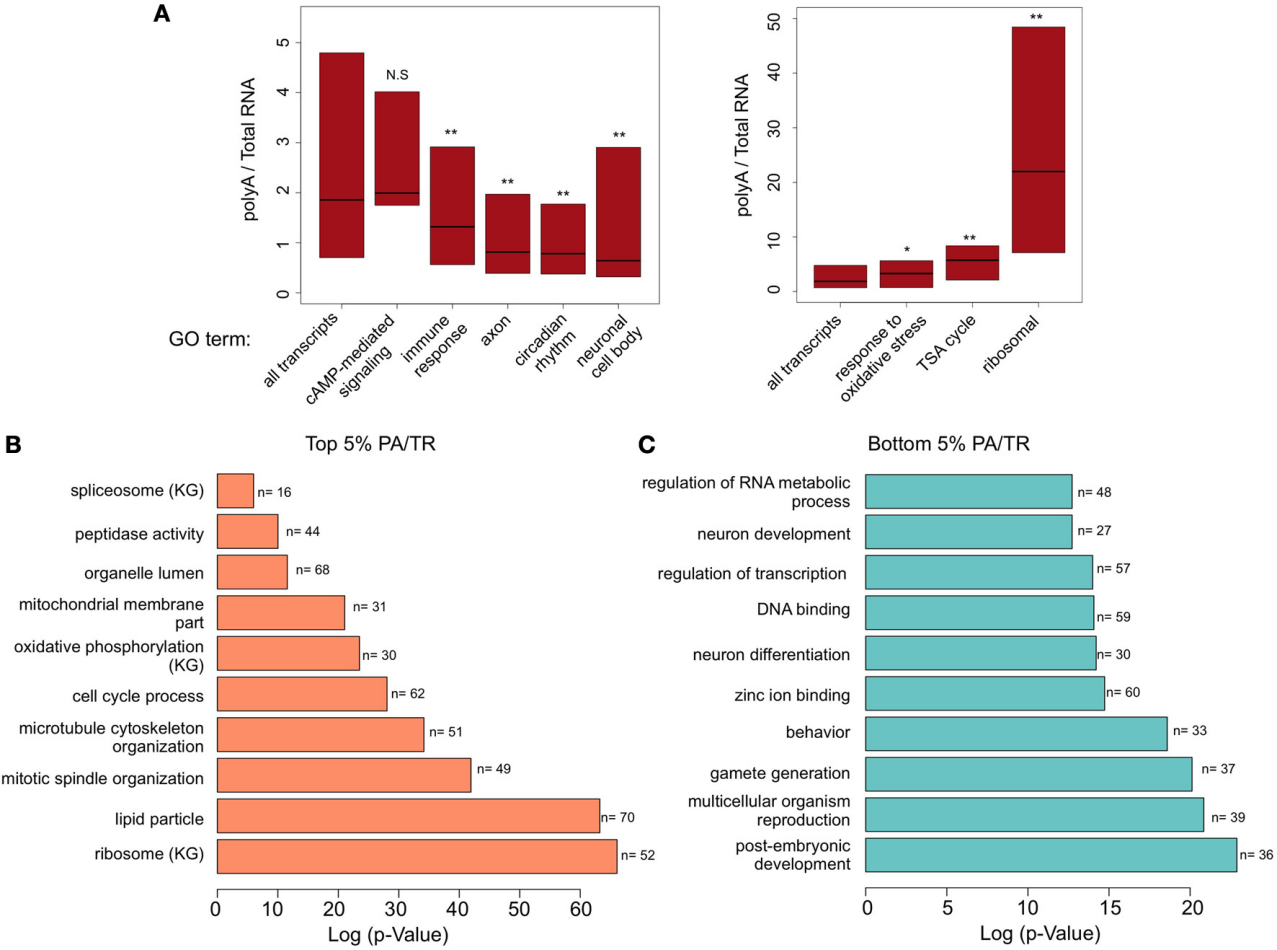
**Gene ontology (GO) enrichment analyses (A) Box plot representation (quartiles and median) of transcripts associated with different GO terms.** Number of transcripts at each group is presented at Figure 1B at the top left panel. Mann-Whitney *U*-test was performed to determine statistical significance of the differences. ^*^p < 0.005; ^**^p < 0.0001. NS, non-significant. **(B)** Results of DAVID functional annotation analysis to examine GO enrichment in genes with the top 5% and **(C)** bottom 5 % PA/TR values. The data presented is log transformed *p*-Value (FDR corrected) of GO terms or KEGG pathways (KG) found to be enriched in the tested group of genes.

Rather than being a direct reflection of mRNA stability, low PA/TR ratios may indicate nuclear retention or specific control of polyA tail length not related to mRNA turnover. In order to test the validity of our approach, we looked at the PA/TR ratio of specific groups of mRNAs that are known to have long or short half-lives (Figures 1B, 2A). We first analyzed mRNAs encoding housekeeping protein. We observed that mRNAs encoding proteins with the GO terms ribosomal and TCA cycle enzymes were significantly enriched in the group of mRNAs with high PA/TR ratios (high stability; *p* = 3.13e^−147^ and *p* = 1.92e^−12^, respectively, Figure 2A). On the other hand, we found circadian-regulated mRNAs among the subset of genes with low PA/TR ratios (*p* = 1.62e^−47^); circadian-regulated mRNAs are by definition short-lived as they display mRNA oscillations and do not accumulate through the day. Therefore, we conclude that our approach can be used to identify differentially stable mRNAs.

### Gene groups in the extreme of the PA/TR distribution belong to specific gene ontology categories

Evaluation of PA/TR values of genes associated with other GO terms gave interesting results. Genes involved in immune response were enriched among the group of genes with low PA/TR ratio (*p* = 7.6e^−9^); genes in the oxidative stress response group had higher PA/TR ratio (*p* = 0.00037) (Figure 2A). Interestingly, genes associated with neuronal-related GO terms such as axon and neuronal cell body were significantly enriched among the mRNAs with low PA/TR ratios (*p* = 3.94e^−43^ and *p* = 1.012e^−18^, respectively), suggesting that mRNAs encoded by genes in this group are under high post-transcriptional regulation (Figure 2A).

To determine which types of mRNAs are in the most stable or unstable groups of genes, we determined the types of transcripts that are particularly enriched in the extremes of the PA/TR distribution. These transcripts should be extremely stable (high PA/TR ratio) or unstable (low PA/TR ratio). We selected the transcripts in the top 5% or bottom 5% of the PA/TR ranking and tested whether these transcripts are enriched for specific GO terms (Figures 2B,C). As expected, transcripts with high PA/TR ratios were enriched for genes with GO terms related to housekeeping functions like ribosomal, enzymes and cytoskeleton organization (Figure 2B). Interestingly, we found that genes encoding proteins involved in cell cycle, luminal proteins, and nuclear mRNA splicing were also enriched in this fraction, suggesting that their mRNAs are long lived (Figure 2B).

In addition, we found that many more GO terms were enriched in mRNAs with low PA/TR ratios (Figure 2C). Notably, genes involved in brain-related processes were highly enriched in the less stable, short-lived mRNA group. These include genes involved in neurological system processes, cognition, sensory perception, behavior, and synapse organization. In addition, genes involved in transcriptional control (such as DNA binding proteins) belonged to the group of short-lived messages. The strong quantitative and qualitative differences between the genes enriched in both extremes of the PA/TR ratio, reinforces the notion that post-transcriptional control is central in brain physiology and function.

**Figure 3:**
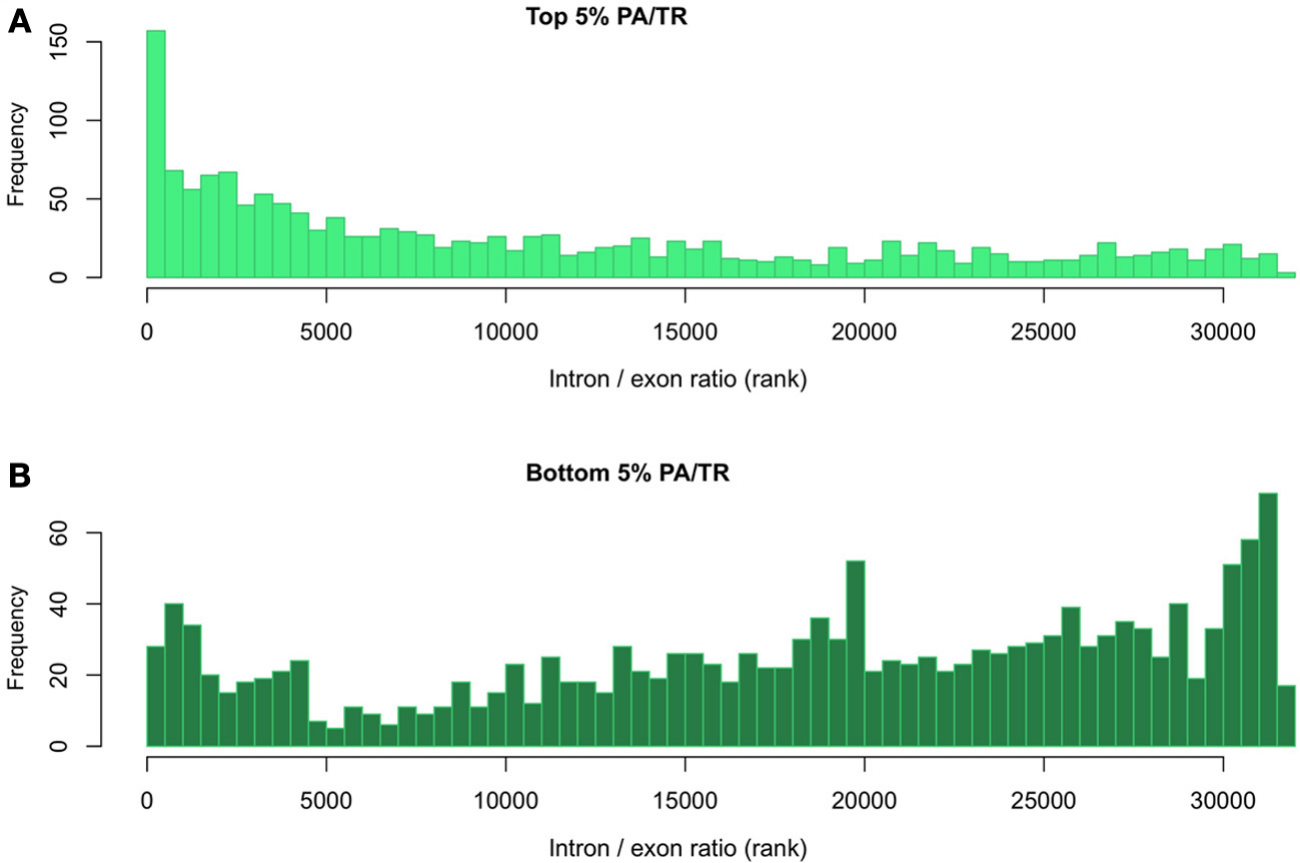
**Evaluation of mRNA stability using the relationship between the intronic and exonic signals. (A)** Distribution of intron/exon ratio of transcripts with top 5% and **(B)** bottom 5% PA/TR values. All transcripts were ranked according to their intron/exon RPKM ratio. The rank values of the top or bottom 5% PA/TR were extracted and plotted.

As stated above, the PA/TR ratio may reflect factors other than mRNA stability. We therefore used an independent measurement to further analyze the genes in the top 5% and bottom 5% of the PA/TR distribution. Intronic data has been used in the past as surrogate of transcription. As the total RNA-seq data includes signal from introns and exons, this dataset can also be used to independently test mRNA stability by calculating the relative amounts of introns and exons for a given mRNA. Hence, we calculated the ratio of intronic vs. exonic signal (I/E) for those genes at the extremes of the PA/TR distribution. We expected that mRNAs with high turnover rates and for which we computed low PA/TR ratios will have high I/E ratios and that those genes in the upper end of the PA/TR distribution would display an opposite trend. In order to avoid misinterpretations of the results due to different scaling factors, we based our comparison on the ranking of the different ratios. We observed that the mRNAs ranked as very stable (top 5%) using the PA/TR ratio were among the transcripts with lowest I/E ratios (less nascent compared to mature mRNA, hence more stable (Figure 3A). In addition, those mRNAs ranked as very unstable in the PA/TR ratio measurement had highest I/E ratio, further validating our approach (Figure 3B).

### PA/TR ratio correlates with transcript abundance only for lowly expressed mRNAs

**Figure 4:**
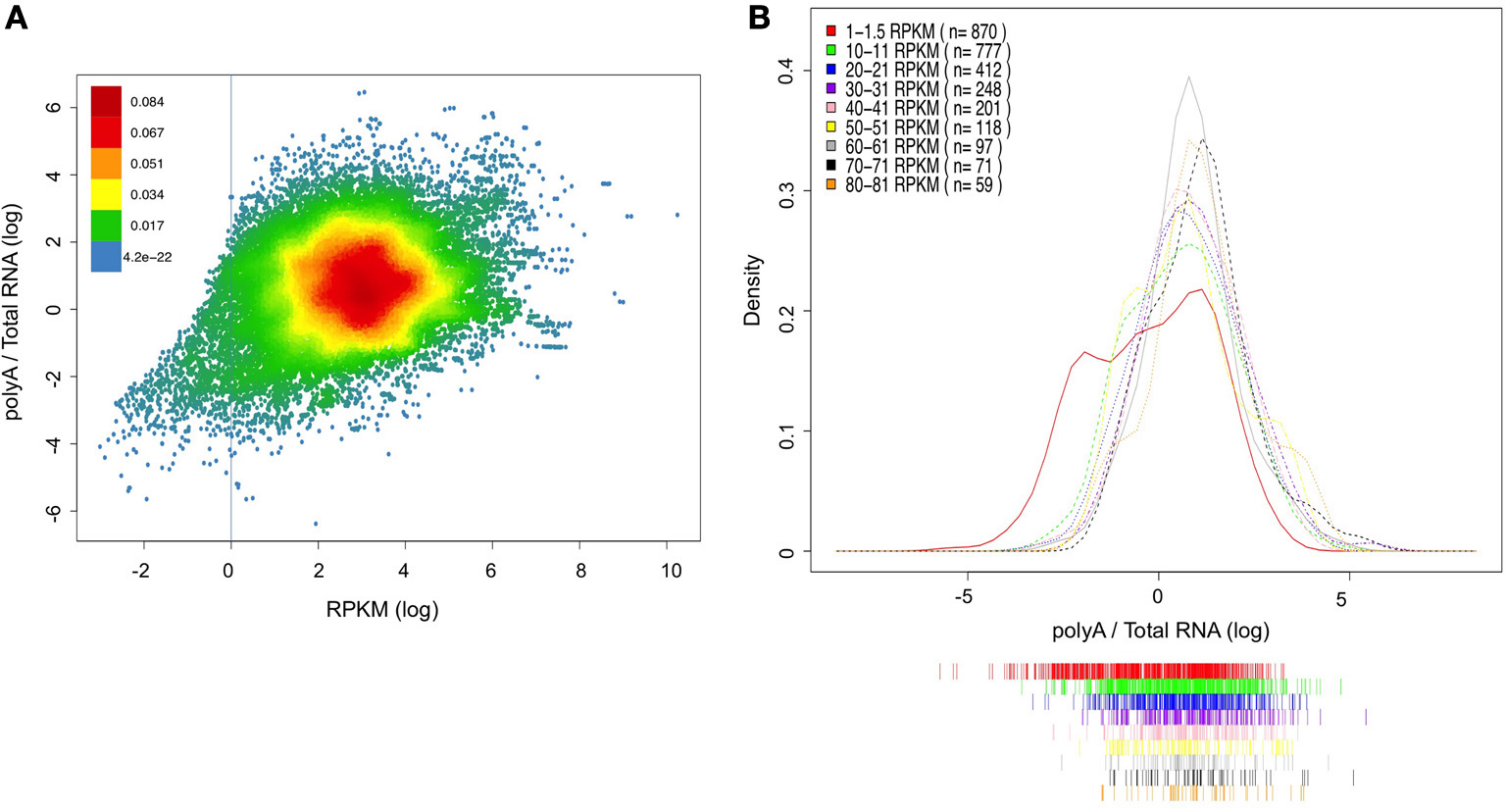
**Association between gene expression and PA/TR ratio. (A)** Correlation between RPKM value of each transcript and its PA/TR ratio. The data is presented as log transformed values and the density of the dots at the plot is represented by the different colors (see color key). Red line represents level of 1 RPKM (log value of 0). **(B)** PA/TR distributions of groups of transcripts with various RPKM values. The RPKM range in each group is indicated at the top left panel. The data is presented as log transformed values.

In order to further validate the ability of the PA/TR ratio to evaluate mRNA stability, we decided to examine whether the PA/TR has any bias for low or high expressed mRNA. For assessing this possibility, we used a linear regression model that takes into account the relationship between transcript expression levels (RPKM values of the poly A selected RNA) and its predicted stability (PA/TR ratio). Indeed, this model show a positive correlation between the mRNA abundance and stability (*n* = 32898, *r* = 0.29, *p* < 0.00001). However, only ~9% of the change in PA/TR ratio can be explained by the expression levels (R-squared = 0.0879) demonstrating that the PA/TR ratios are not a mere reflection of mRNA abundance. Moreover, when filtering out the very low expressed mRNAs (those expressed less than 1 RPKM), the explained fraction is reduced to only 3.5% (*n* = 31482, R-squared = 0.0346). Interestingly, for the lowly expressed transcripts, the explained fraction is more than 30% (*n* = 1416, R-squared = 0.3181) Figure 4A shows a scatter plot of the correlation (the red line represents RPKM value of 1).

In order to look in more detail into the relationship between the PA/TR ratio and mRNA abundance, we selected groups of transcripts based on their expression levels (e.g., 1–1.5, 10–11, 20–21 until 80–81 RPKM) and compare their PA/TR ratio distribution (Figure 4B). ANOVA test demonstrated that there is no significant difference in the distribution of PA/TR values across the range of 20-80 RPKM (*p* = 0.09), showing that in this range, transcripts with four times difference in expression levels can have the same PA/TR ratio. Indeed, only the two groups with lower expression (RPKM 1–1.5 and 10–11) showed significantly different distribution, as they are clearly enriched for transcripts with low PA/TR ratio (*p* < 0.0001 for both). These results demonstrate that PA/TR ratio does not correlate with transcripts abundance globally. However, transcripts with very low mRNA abundance have in average lower PA/TR ratio, but we favor the interpretation that this is a result rather than a bias of the PA/TR ratio (see discussion).

### Global assessment of miRNA abundance in the *drosophila* brain

**Figure 5:**
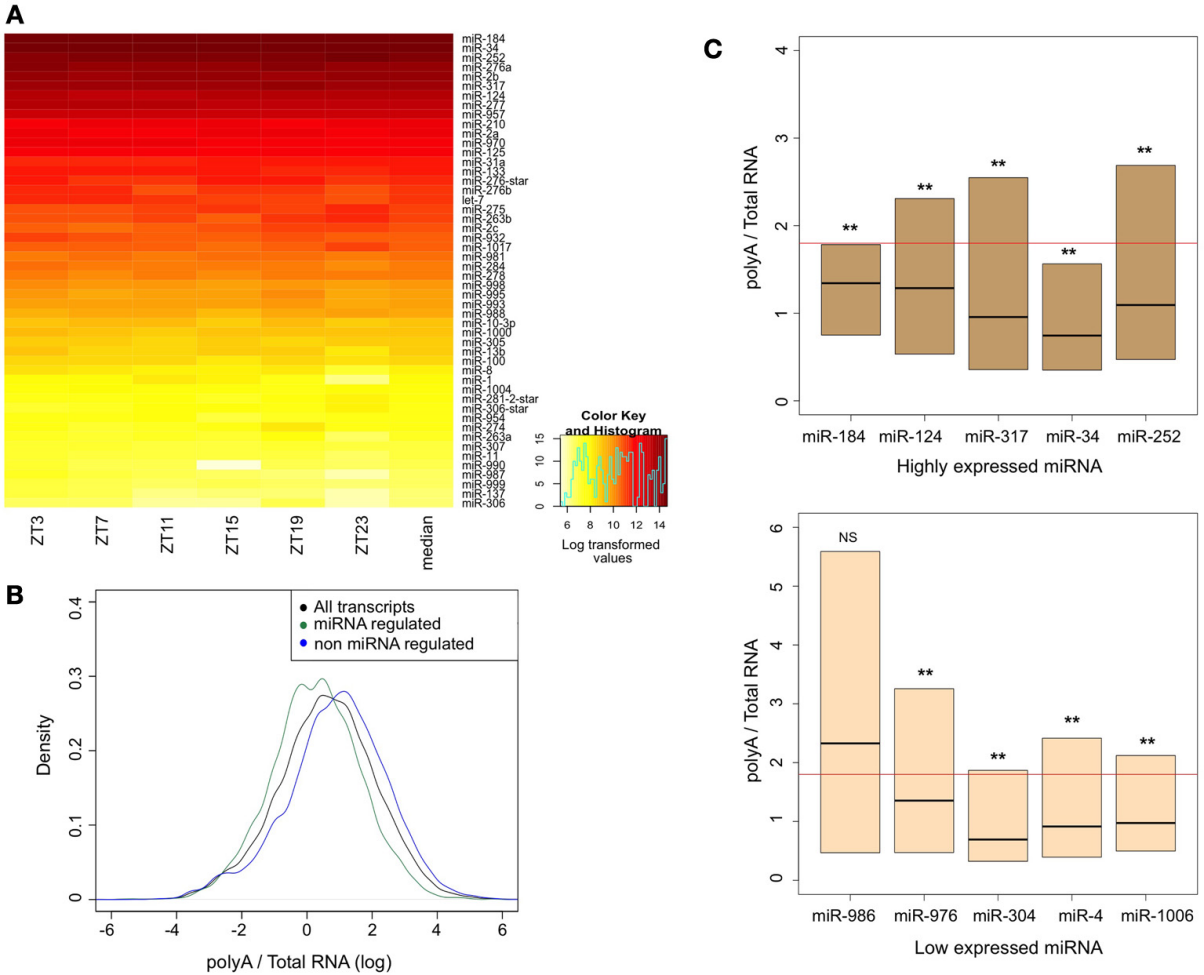
**Assessment of miRNA expression in the brain and the stability of their targets. (A)** Heat-map representation of top 50 miRNA expressed in the *Drosophila* brain. Fly brains were collected across six time points of the circadian day (ZT, zeitgeber time). RNA was extracted and loaded to Affymetrix array chips. miRNA expression levels were averaged across the six time points for further analysis. **(B)** Density plot comparing the distribution of polyA/total-RNA values for all transcripts (black line; *n* = 32898) with those of miRNA-regulated genes (green line; *n* = 15206) and non miRNA-regulated genes (blue line; *n* = 16,456). Data was log-transformed to achieve normal distribution. Mann-Whitney *U*-test and bootstrapping approach (10,000 bootstrap samples) showed significant difference between the groups (*p* < 0.0001). **(C)** Box plot representation (quartiles and median) of PA/TR values of different miRNA target genes. For each list of miRNA targets, Mann-Whitney *U*-test was used to determine statistical significance of the differences. Numbers of transcripts at each group are summarized at Supplementary Table 2. Horizontal line represents the median values for all transcripts. ^**^p < 0.0001. NS, no significant.

Our meta-analysis revealed that several types of mRNAs are highly regulated at the post-transcriptional level. MiRNAs or RNA binding proteins could mediate this regulation. Since there is no publicly available genome-wide expression data available for miRNAs in the *Drosophila* brain, we generated our own dataset. We purified RNA from dissected brains and determined the abundance of individual miRNAs using oligonucleotide microarrays. In order to minimize effects due to the time of collection, we isolated RNA from brains of flies collected at six different times of the days. Figure 5A shows heat-map representation of top 50 miRNA expressed in the *Drosophila* brain. miRNA expression levels were averaged across the six time points for further analysis.

### Transcripts with low PA/TR ratios are enriched for miRNA binding sites, but their stability is not correlated to the abundance of the predicted regulatory miRNA

In order to test whether miRNA-mediated regulation has a major impact on processes in the brain, we tested whether the less stable mRNAs were enriched for predicted miRNA targets. We used dataset of predicted targets of conserved miRNA families (using TargetScanFly) and estimated the PA/TR ratio of these transcripts. We found that those mRNAs which have been predicted to be regulated by miRNA (*n* = 15206) are enriched among the less stable transcripts. Mann-whitney *U*-test and bootstrapping approach (10,000 bootstrap samples) showed that the difference is statistically significant (*p* < 0.0001). This demonstrates a key role for miRNAs in regulating mRNA stability in the fly brain (compare the distribution of the PA/TR ratios for all mRNAs and those that have been predicted or not to be miRNA-regulated in Figure 5B).

Last, we tested whether there is a correlation between miRNA expression levels in the brain and the stability (calculated from the PA/TR ratio) of their target mRNAs. We divided miRNAs into groups based on their expression levels. For each miRNA we calculated the PA/TR ratio of its predicted targets and tested for significant differences between its targets values and the entire transcript population. For almost all the miRNAs, their predicted targets had significantly lower PA/TR values than the entire transcript population: Out of 94 miRNA families only seven were not found in the group with lower PA/TR targets (Supplementary Table 2). Surprisingly, we did not find any correlation between the expression levels of the miRNAs and the PA/TR ratio. Predicted targets of both highly expressed and lowly expressed miRNA had low PA/TR ratio (Figure 5C), and applying Spearman's correlation test did not show significant correlation between miRNA expression and PA/TR values (*p* = 0.109). These results demonstrate that although miRNA regulation is a key regulatory mechanism in the brain, there is a complex, non-linear correlation, between transcripts containing miRNA target sequences and miRNA expression levels.

## Discussion

In this work we utilized previously published RNA-seq data and newly generated brain-specific miRNA expression data to globally estimate mRNA turnover rates in the *Drosophila* brain and to evaluate the mechanism behind this regulation. In order to estimate globally mRNA turnover rates, we compared the levels of each transcript in polyA+ purified and rRNA-depleted RNA samples. More specifically, we generated a PA/TR ratio that should directly correlate with the extent of post-transcriptional control and inversely with mRNA stability. We validated our approach by showing that mRNAs known to be highly stable like those encoding proteins related to the ribosome and cytoskeleton function, have a high PA/TR ratio. At the opposite end of the stability spectrum, mRNAs known to have high turnover rates like those encoding synaptic, circadian, and other proteins display PA/TR ratios indicative of short half-lives. Interestingly we found that mRNAs encoding proteins involved in key neuronal functions are among the most highly regulated mRNAs at the post-transcriptional level. MiRNAs seem to play a key part in mRNA stability in the brain, as transcripts with very low PA/TR ratio are strongly enriched for miRNA binding sites. However, miRNA regulation is likely to be complex and redundant, as we did not find correlation between miRNA levels and the PA/TR ratio of their predicted mRNA targets in the brain.

Although we have validated our strategy, we acknowledge that it provides an indirect measure of mRNA stability. This is because the PA/TR ratio may reflect nuclear retention, inefficient splicing, and other modes of regulation like cytoplasmic polyadenylation instead of mRNA turnover. However, we believe that it can be certainly assured that genes with low PA/TR ratio are under strong post-transcriptional control. Indeed, modes of post-transcriptional regulation that does not lead to mRNA decay (e.g., cytoplasmic polyadenylation) could constitute an important point of control for certain mRNAs like those that are translated in synapses. It is well known that synaptic-translated mRNAs are associated with the miRNA machinery and specific RNA binding proteins until their translation. The PA/TR ratio thus measures more generally the extent of post-transcriptional control of mRNA levels rather than being a measure of mRNA turnover.

We found that for most transcripts, there is no correlation between their expression and their stability measured by the PA/TR ratio (Figures 4A,B). However, we found that very lowly express genes are among the less stable mRNAs. We don't believe that this is the result of bias in the analysis or calculation of the PA/TR coefficient but rather a biological meaningful result. In other words, we believe that our results indicate that lowly expressed genes are the result of not so low expression coupled to high mRNA turnover. This could be a way to diminish gene expression noise, as it is known that lowly transcribed genes are subjected to high expression noise. Indeed middle transcription followed by strong post-transcriptional control has been proposed to be an efficient way to generate low mRNA levels without much noise (Hornstein and Shomron, 2006). Given the key function of the genes with low expression in the brain, this seems a fair tradeoff. It should be pointed out that the data we utilized for this study is extremely deep (~20 million reads per sample for polyA selected RNA, and ~40 million paired-end reads per sample for non-polyA), so even the very low genes are well represented (in terms of total amounts of reads) in the PA samples, therefore, we don't think that our PA/TR ratio has diminished performance in this extreme of the expression distribution.

Although our results suggest a key role for miRNAs in post-transcriptional control, we were surprised to find that there is no correlation between the levels of brain miRNAs and the extent of post-transcriptional control of their predicted targets. This could be due to several factors. First, it is known that mRNAs are usually targeted by several miRNA species, with certain miRNAs expressed in some cell types but not others (Bartel, 2009). Second, miRNA abundance is not always reflective of the functional activity. Indeed, sequencing of AGO-1 associated (RISC-bound) miRNAs is a more accurate measurement of the abundance of functional miRNAs as only a fraction of miRNAs present in a cell are incorporated into a RISC and are thus functional at a given time (Krol et al., 2010). Third, our correlations are based on miRNA-target predictions. Although algorithms like Target-Scan usually display low false positive rates, many meaningful interactions might be missed (Yue et al., 2009). Hence the lack of correlation could be due to failure in the miRNA-target prediction algorithm, although we feel that this is unlikely as we observed the lack of correlation using only the evolutionary conserved miRNAs. Last, as the brain is highly heterogeneous in neuronal cell types, it is possible that miRNAs expressed at very low levels globally have key functions in specific neuronal groups. A last consideration is that our approach does not consider expression levels. Two genes with equal PA/TR ratios but very different expression levels may respond very differently to a given miRNA. Based on this consideration, we believe that identification and analysis of a dataset of AGO-1-associated mRNAs and miRNAs would shed additional light on post-transcriptional regulation in the brain (Varghese et al., 2010; Aw and Cohen, 2012; Weng and Cohen, 2012).

In sum, our comparison of levels of total or polyA-selected RNA allowed us to evaluate the extent of post-transcriptional control for all brain-expressed mRNAs. The lack of a strong correlation between the expression levels of miRNAs in the brain and the stability of their target mRNAs indicates that much remains to be learned about the modulation of brain-specific mRNAs in the fly. Our work provides a valid approach for analysis of mRNA stability and indicates a central role for miRNAs in regulating mRNA levels in the brain.

## Materials and methods

### RNA-seq data analysis

We used RNA-seq data published by Hughes et al., available at GEO (accession number: GSE29972) (Hughes et al., 2012). In this paper, the authors generated RNA libraries with polyA selected (PA) or ribosomal-depleted RNA (TR). Our analysis was based on the processed data published by the authors, which includes RPKM values calculated as described. Two replicates of CS samples from ZT0 and ZT12, both polyA selected and ribo-depleted, were used for the analysis. Non-coding genes and lowly expressed transcripts (bottom 20% RPKM values) were filtered out. For each transcript, we divided polyA RPKM values with non-polyA RPKM values of the corresponding sample to determinate PA/TR ratio. The average of PA/TR values of both replicates and time points was used for further analyses. To determine Intron/Exon (I/E) ratio we divided the average exonic and intronic RPKM values and the data was ranked prior to comparison to PA/TR ratio. PA/TR data was log-transform to achieve normal distribution for data visualization and prior to applying linear regression model. All data used in this study is included in Supplementary Table 1 (see also Figure 1). All analyses described in this paper were performed using R version 3.0.1.

### Gene ontology enrichment analysis

Gene Ontology database (http://www.geneontology.org/) was used to obtain lists of genes associate with different GO terms. For each list of genes, we extracted PA/TR values of the transcripts, calculated median and used the non-parametric Mann-Whitney *U*-test to determine statistical significance. DAVID functional annotation tool (http://david.abcc.ncifcrf.gov/home.jsp) (Huang et al., 2009) was used to examine GO enrichment in the groups of genes with top 5% or bottom 5% PA/TR values. The list of expressed transcripts (top 80% RPKM values) was used as the background in the analysis. The data presented is log transformed *p*-Values after FDR correction.

### *drosophila's* brain sample preparation

For profiling the expression of miRNA, 3–5 days old *Drosophila.M* Canton-S flies were entrained in 12:12 LD cycles. Fly brains were collected across six time points of the circadian day. At each time point twenty five brains were dissected, and completely cleaned from trachea and fat tissue, in ice cold PBSX1. Brains collected into an eppendorf were immediately immersed in Lysis/Binding buffer (Ambion, AM1560) and kept on ice for the rest of the dissection. By the end of each dissection round brains were homogenized using a rotor blade and frozen in liquid nitrogen.

### RNA extraction

Extraction of small RNA containing-total RNA was performed using the mirVana miRNA isolation kit (Ambion, AM1560). Organic extraction using Acid-Phenol:Chloroform was done according to the manufacture's protocol. Following elution samples were treated with TURBO DNase (Ambion, AM2238) according to the manufacture's protocol. Finally, RNA was recovered by isopropanol precipitation supplemented with glycerol.

### Affymetrix genechip miRNA 2.0 array

Pre-miRNA and mature miRNA expression levels were studied using Affymetrix GeneChip miRNA 2.0 Array. 600ng from each of the miRNA containing-total RNA were loaded to six array chips. Affymetrix Expression ConsoleTM Software was used to normalize and calculate summary values from Affymetrix CEL files. Data were background-corrected by the RMA method. Heatmap was generated using the heatmap.2 function of the gplots package in R. miRNA expression levels were averaged across the six time points for correlation analysis.

### miRNA target genes analysis

List of conserved miRNA families and their targets was obtained from TargetScanFly (http://www.targetscan.org/fly/). PA/TR values of miRNA target genes were extracted and the median for each miRNA targets group was calculated. Mann-Whitney *U*-test was used to estimate statistical significant comparing to all transcript population and the *p*-Values were FDR corrected. For estimating the significance of the differences between all miRNA targets and non-miRNA targets PA/TR values bootstrapping approach was also applied (10,000 bootstrap samples). Spearman correlation test was used to examine relationship between miRNA expression levels and PA/TR value of their targets.

## Author contributions

Shaul Mezan: Performed the experimental work and helped with the writing of the manuscript. Reut Ashwal-Fluss: Lead the analysis of the data and helped with the writing of the manuscript. Rom Shenhav and Manuel Garber: Helped with the analysis of the data. Sebastian Kadener: Designed the experimental and guided the analytical part. Wrote the manuscript.

### Conflict of interest statement

The authors declare that the research was conducted in the absence of any commercial or financial relationships that could be construed as a potential conflict of interest.
